# Ability of salivary biomarkers in the prognostic of systemic and buccal inflammation

**DOI:** 10.4317/jced.53776

**Published:** 2017-05-01

**Authors:** Aida Gutiérrez-Corrales, Elena Campano-Cuevas, Gabriel Castillo-Dalí, Daniel Torres-Lagares, José-Luis Gutiérrez-Pérez

**Affiliations:** 1 Stomatology Department. Faculty of Dentistry. Seville, Spain

## Abstract

Nowadays, there is a growing interest in using saliva as an alternative sample for the diagnosis, prediction and progression of several diseases. It has been established that some molecules found in saliva are related to oral inflammatory processes and systemic health status. Furthermore, it is known that saliva is crucial for the carrying out of different functions in the oral cavity and its role in the local modulation of inflammatory and immune response is being thoroughly studied by the health research community. The aim of this review is to analyze the most important biomarkers which have been utilized in biomedicine during the last two decades in order to establish a correlation between certain specific salivary biomarkers and systemic inflammation. Then, we discuss the utility of total proteins, immunoglobulin A and alpha-amylase as biomarkers for the prognostic of local inflammation after oral surgery.

** Key words:**Inflammation, salivary biomarkers, systemic disease, buccal surgery, total proteins, inmunoglobulin A, Alpha-amylase.

## Introduction

During the last decades, salivary diagnostic approaches have been developed to diagnose and monitor oral diseases such as caries risk, microbial sepsis, inflammation and genetic pathologies like oral tumors and cysts. Buccal inflammatory biomarkers are biomolecules as proteins and derivatives which show an increase or decrease in buccal processes coursing with inflammation. These biomarkers are commonly used in biomedicine to confirm the level and type of inflammation present in oral diseases. The local concentration of these proteins varies depending on habits, age, sex or the place where the sample was taken from (oral mucosa or another). The most common fluids used in inflammation prognostic assays are: Saliva, crevicular fluid and serum. Cytokines (TNF-α & IFN-γ, IL-1,4,6,8,10), IgA, α-Amylase, Cortisol and Total Proteins

are the most frequently used inflammatory salivary biomarkers in the recent decades and utilized in biomedical prognostic (1-5).

In the brain, cytokines are essential components of a signaling complex network in body inflammation. IL-1, IL-4, IL-6, IL-8 e IL-2 along with TNF- α and the IFN-γ are the most habitual cytokines used like inflammatory salivary biomarkers since they have become in the most influential molecules in the risk detection site of the patient, also for other inflammatory chain factors activation and for the proper inflammation process (6-9). In addition, the alpha tumor necrosis factor is a very important protein cytokine for neutrophils and other blood cell activation in acute inflammatory process into sepsis and oral diseases like periodontitis and its diagnosis (6,10). Furthermore, the Gamma interferon is also a protein cytokine that is secreted by T-cells to perform macrophages activation in oral diseases like periodontitis in the inflammation process (11).

Focused on IgA, it is an immunoglobulin type protein like a biomarker of immunological activity which is presented in seromu-cinous fluids as the primary defense against pathogens. The level of this protein increases significantly in case of infectious diseases, as well as IgG and IgM such in case of periodontitis, showing a positive correlation between the severity of periodontal, gin-gival damage and IgA concentrations. These concentrations are crucial in antibacterial, antifungal and antiviral process (12-17).

The alpha amylase is an enzyme of polysaccharides degradation which is used on the treatment and in the prognostic of inflammatory diseases, in actinomycetems infection and against some bacteria as the ones that cause caries (3,18-22). The AASH, especially for its high concentration, has been used as a biomarker. Its concentration is 1080 ± 135.6 IU or 476 ± 120 mg / ml in adults.

Nevertheless, all the proteins concentration is the sum of the levels of each protein into the oral fluids and they are also quite relevant and significant in inflammation process, in clinical analysis and in the diagnostic of some diseases which show a linear increase correlation in patients with high advanced age and caries (12,23). However, cortisol is an anti-inflammatory hormone that is used in several assays but its results present several variances between sex, day and type of assay and they are not always statistically significant (4,12,20). Others buccal salivary biomarkers are involved in oral fluids: Protein-2, Protein-3B, Chaperokines, Cystatin, Lysozime-C, Lipocalin, BPI, Collagenases MMP-8, PLUNC, Mucins, Peroxidases, Prolin-rich Protein, Chromogranine-A, LDH, Reactive Protein-C (18-20,24,25).

The advantage of using saliva as a diagnostic means is that their sampling is easy and non-invasive but this potential has not been fully exploited, due to the lack of some techniques to detect components that are in less concentration. However, it is expected that with the advancement of bioinformatics, genomics and proteomics, saliva becomes increasingly a study tool, due to its ability to reflect conditions of oral and systemic health (26,27).

It is obvious that after any surgery in the oral cavity, it appears in varying degrees postoperative swelling as physiological response of the organism; the preventive philosophy of such symptoms is based on anticipating to the appearance for trying to minimize them. While postoperative orofacial pain is a direct result of the damage caused by the surgery and the body’s response to it, we should not forget that pain perception varies with each patient based on certain factors. The importance of the anxiety and the degree of stress of the patient in the postoperative pain perception, has been widely studied by different authors, who defined that anxiety can lengthen the time of the intervention by inducing greater pain and inflammation and it can also increase the intensity of both postoperative symptoms possibly by reducing the threshold of pain tolerance (28).

Saliva concentrations of total protein, immunoglobulin A and alpha-amylase vary when inflammation occurs within infectious processes of the oral cavity. Similarly we seek to relate the preoperative values of those salivary markers with the postoperative ones, which will allow us to establish a prognostic value of inflammation that will occur in the postoperative period. Through the availability of studies to increase knowledge of the salivary prognostic factors related to inflammation will help to improve the quality of the healthcare. Furthermore, this study proposes innovative cooperation of two areas of health sciences: oral surgery and molecular biology (29).

## Discussion

In recent years, several analytes in saliva have been evaluated as biomarkers for many diseases including: cancer (30,31), type II diabetes (32), infections (33,34), pulmonary (35) and cardiovascular diseases (36), among others. These findings allow facilitating the diagnostic of systemic illness because saliva is a non-invasive and easy-to-use sample.

Abnormal cortisol rhythms before epithelial ovarian cancer treatment has been associated with higher inflammation in the tumor zone and decreased survival of patients. In this sense, nocturnal concenrations level has been proposed as a measure of the hypothalamic-pituitary-adrenal (HPA) dysfunction that occurs in this kind of cancer and an indicator of disease severity. In this study, an association between ascites pro-inflammatory cytokine IL-6 and salivary cortisol was observed; indicating night concentration may be an efficient biomarker for measuring HPA function in ovarian cancer population (37).

Other researchers have focused on secretory proteins as cancer biomarkers because they are closely related with the malignant tumor angiogenesis, differentiation, invasion and metastasis process (38). Although most studies have been performed to identify such biomarkers in serum (38-40), there are some of them interested in identifying saliva secretory proteins that could be used as cancer biomarkers. Shiiki et al. have examined levels of Prostate-specific Antigen (PSA) in saliva and its association with serum PSA in two groups of patients with prostate adenocarcinoma (PA), a low-serum PSA concentration group and a high-serum PSA concentration group with high risk of recurrence or metastasis. They concluded that saliva PSA may be a useful PA biomarker in those cases in which patients have recurrent or metastatic PA, as in this situation saliva PSA concentration is associated with blood PSA (41). In this same field, Streckfus et al. suggest salivary proteins as biomarkers for carcinoma in situ of the breast (DCIS). They studied saliva specimens from three groups of women (ten healthy subjects, ten patients diagnosed with a benign breast tumor and ten women diagnosed with DCIS). Their results revealed that protein-by-products of oncogenic expression appear in the saliva of breast cancer patients but not in healthy controls (42).

In the other hand, there are several antigens that are expressed in tumor cells but not in normal ones or, at least, not at that high concentration so these tumors associated antigens might act as tumor associated antigens. In this situation, the immune system would prepare for recognizing tumor cells (43). At this point, it is reasonable to think that several antibodies can act as predictors in the diagnosis of this malignancy and, in fact, protein p53 antibodies IgA and IgG have been found in the serum and saliva of patients who overexpressed p53 in their tumor tissues, demonstrating that the detection of these antibodies could identify oral squamous cell carcinoma with p53 mutations, which occurs in 50% of cases (44,45).

In coronary heart disease, several biomarkers can be detected in saliva and its levels vary from those measured in healthy individuals. In this sense, changes in concentration of active matrix metalloproteinase (MMP)-8, creatinine phosphokinase, troponin I have been observed in saliva and these altered concentrations of biomarkers correlates with serum concentration, so again saliva results a useful sample for diagnosing systematic diseases (46-48).

In Acute Myocardial Infarction (AMI), fast diagnostic is essential for optimal therapy to be offered because every minute of delay in treating AMI increases the mortality rate (49). Saliva contains thousands of biomolecules that are derived from the local capillary bed (50), which makes saliva a potential source for a rapid diagnosis of AMI in the emergency setting (51-53). In the research of specific salivary biomarkers with clinical potential for the assessment of AMI, C-reactive protein, sCD40L and CK-MB, as well as creatinine phosphokinase, TnT, and TnI resulted useful diabetes (32). 

As well as others systemic diseases, inflammation is a condition in which many studies have focused. Among them a small number of studies have investigated inflammation markers in saliva as they are altered in response to an acute stressor. In this sense, several studies have proposed salivary biomarkers to identify different stress status. Among these, salivary levels of pro-inflammatory cytokines and anti-inflammatory cytokines have resulted to be increased in those subjects exposed to a stress situation (54-56).

In this field, Mahmood and Ibrahim, 2013, have demonstrated increased levels of pro-inflammatory interleukin 1-beta in saliva from 24 healthy students when they were subject to examination stress. These results support those found by Usui et al., 2012, when testing saliva from 10 physically active participants exposed to a 60 minute exercise stressor. In this case, levels of IL-1b, IL-6 and TNF-alpha were significantly higher during completion than those measured during the resting session. Besides, elevation in TNF-alpha and IL-2 salivary levels have been detected in University professors 120 after exposures to social-cognitive stressors (56). The same approach was observed when testing concentrations of anti-inflammatory cytokines IL-4, in saliva samples taken from individuals before and after stress conditions.

Others biomarkers have been assayed not because it is useful as inflammation indicators, but as direct markers of immune system status. This way, salivary immunoglobulin A (sIgA) has been measured as a predictor for immunity variations caused by stress (57).

The immune response has been proposed as a mechanism of protection, a mediator of injury, and a need for repairing tissues in periodontal disease. Immunoglobulins have been recognized as elements in human inflamed gingiva. Brandtzaeg and Kraus studied the most striking difference between saliva samples in healthy and inflamed gums and it was found a predominant increase in the number of plasma cells containing IgA using the immunofluorescence method (58).

Many studies have been published about the concentration of IgA in saliva but data are extremely confusing. The concentration of IgA in samples of whole saliva or mainly of the parotid (59), has been discussed in different investigations, linking them with oral pathological processes, obtaining different results due to the influence of various factors such as the maintenance in cold, saliva collected at rest or after some kind of stimulation, or stress to which the patient has been undergone. Several studies about salivary IgA concentration was analyzed in young patients with oral inflammation to see if there were significant differences between saliva samples collected at rest or stimulated (60). According to scientific evidence, the IgA concentrations is higher in parotid than mixed saliva and after stimulation, the concentration of salivary IgA decreases in both types of saliva (3.1 ± 2.2 mg / dl in mixed saliva and 6.8 ± 5 mg dl parotid salivary).

Similarly, another study measured the concentration of IgA in mixed saliva and of the parotid exclusively (61) in individuals with gingival inflammation as a consequence of an infection, obtaining in both samples a similarity in the concentration of IgA. This could be due to prolonged activity of bacterial antigens, the stimulation of IgA production, the increase of absorption of combined antigen through inflamed gingiva or glandular local production of IgA among other factors (58).

In addition, investigation has been interested in the use of salivary biomarkers for detecting type-2 Diabetes. Rao et al., 2009, used whole saliva samples from control and type-2 diabetic individuals to identify such biomarkers. They found 65 proteins that were greater than 2-fold more abundant in type-2 diabetes samples compared to control specimens, providing a view of saliva potential utility in detecting and monitoring 

In recent decades, there has been considerable progress in the understanding of the genetic and biochemical properties of the major salivary proteins. Salivary proteins and glycoproteins are the major components of the biofilm, whose function is to keep moisture and act as a barrier to protect the teeth and structures around the teeth (23).

There are numerous defense proteins in saliva. Although some of these molecules are present in very low concentrations, its effects are additive and synergistic, resulting in an efficient molecular defense network of the oral cavity. Moreover, the local concentrations of these proteins near mucosal surfaces (transudate mucosa), periodontal groove (fluid crevicular gingiva) and oral wounds and ulcers (transudate) can be much larger, and often reinforced by immune reactions and inflammatory diseases of the oral mucosa (62,63). Some defense proteins, such as salivary immunoglobulin are involved in innate and acquired immunity. Cationic peptides and other defense proteins such as lysozyme, salivary amylase, cystatins, mucin, peroxidases, and statherin, among others, are primarily responsible for innate immunity. Salivary flow and concentration of various inorganic and organic components may vary by state of health, age, medication, etc (64).

It was observed some alterations in the biochemical composition of saliva in patients with oral leukoplakia and cancer which could be attributed to salivary gland dysfunction caused by alcohol and snuff due to chronic alcohol intake is associated with significant changes in the secretion the parotid saliva and its composition. The alcohol consumption causes a decrease in the flow of whole unstimulated saliva and therefore there is also a lower total protein content, alpha-amylase and electrolytes (63).

The human salivary α-amylase (AASH) is the saliva protein found in greater concentration and has enzymatic activity, which catalyzes the links α-1, 4-glycosidic of starches and carbohydrates. Also it plays an important role in the colonization and metabolism of the bacteria that lead to plaque formation. In solution, this protein binds with high affinity to a select group of oral streptococci, which can assist in debugging or bacterial cleaning the oral cavity (63). It is produced locally in salivary gland and its secretion is controlled by the autonomic nervous system, which has been proposed as a biomarker for the activity of this system. In general, direct measurement of its activity in the oral cavity has allowed its partnership with states of pain, stress and disease. As noted above, the presence in the saliva of endogenous substances in human serum allows saliva be potentially used to diagnose some diseases like oral cancer and breast cancer, periodontal disease, Sjögren’s syndrome, among others (65).

The great variability concentration of salivary proteins has been used to characterize the state of disease of some individuals and is known as biomarkers. According to the National Institute of Health in the United States, this term is given to a quantitatively measurable biological parameter that serves as an indicator of the health and physiological assessments related to pathogenic processes, environmental exposure and the diagnosis of disease or response to drug therapy or therapeutic intervention (66).

Currently, the third molar surgery is the most common surgical technique performed in the oral cavity. This often triggers an inflammatory process which is characterized by symptoms as pain of the operated area, soft tissue swelling with subsequent facial deformity, and sometimes a degree of lockjaw associated. Normally, postoperative requires an average of seven days of labor inactivity primarily associated with the inflammation that occurs. Therefore, trying to minimize any secondary symptoms after oral surgery without interfering in the physiological process of inflammation following surgical trauma should be a primary objective.

These symptoms are terribly uncomfortable for the patient, depending on the intensity of multiple factors such as the complexity of the surgery, the duration, the surgeon’s technique, the existence of iatrogenic, etc. The minimization of such manifestations directly affects the satisfaction of the treatment, improves the life quality of the patient and also reduces the fear to these surgical interventions. The concepts to control postoperative symptoms have changed substantially over the last years as it has advanced the understanding of the pathophysiological basis of pain and inflammation as well as the mechanism of action and pharmacodynamics of analgesics and anti-inflammatory used in their treatment although nowadays there is more emphasis on the importance of preventing both pain and inflammation (67,68).

On the one hand, Holland (69) and Puche *et al.* (70) defend that the degree of postoperative facial swelling is unpredictable and it depends on the different response of the patient to the surgical trauma. Seymour *et al.* (71) and Meechan and Seymour (72) argue that the severity of pain varies from patient to patient and it does not seem to be related with the degree of impaction and surgical trauma required to remove the tooth. On the other hand, Capuzzi *et al.* (73), who claim that postoperative inflammation is predictable because it depends mainly on surgical aggression among other factors.

Due to understand the way of utilize these biomarkers, we presents above the most common material and methods used to detect and measure biomarkers in saliva:

- ELISA 

- Bioplex-Luminex

- FC (Flow Citometry)

- BRADFORD

- MRD 

- IFD

- MANCINI

- LORRY

- MS (Mass Spectrometry) 

All the same, the enzyme immunoassay (ELISA), direct immunofluorescence and flow cytometry (CF) are the most common methods used in molecular biology for prognostic analysis. In this sense, samples can be diluted and resuspended or not in the biomedical laboratory after homogenized by vortex. Then, it is possible to make an ELISA immunoassay using a 96 wells plate to proceed the samples analyze using a plate lector which usually get a standard curve in order to interpolate results currently and finally noise balanced using the “zero” controls and placebo. Looking to a future point of view, salivary fluids seems to be a new efficient diagnose and prognostic method in biomedicine and oral diseases at early state. It is also necessary to remark the new advanced technique in biomarkers measurement; The Detection Chips, which bring the possibility of analyze million samples simultaneously and more analytes per sample ( Table 1).

**Table 1:**
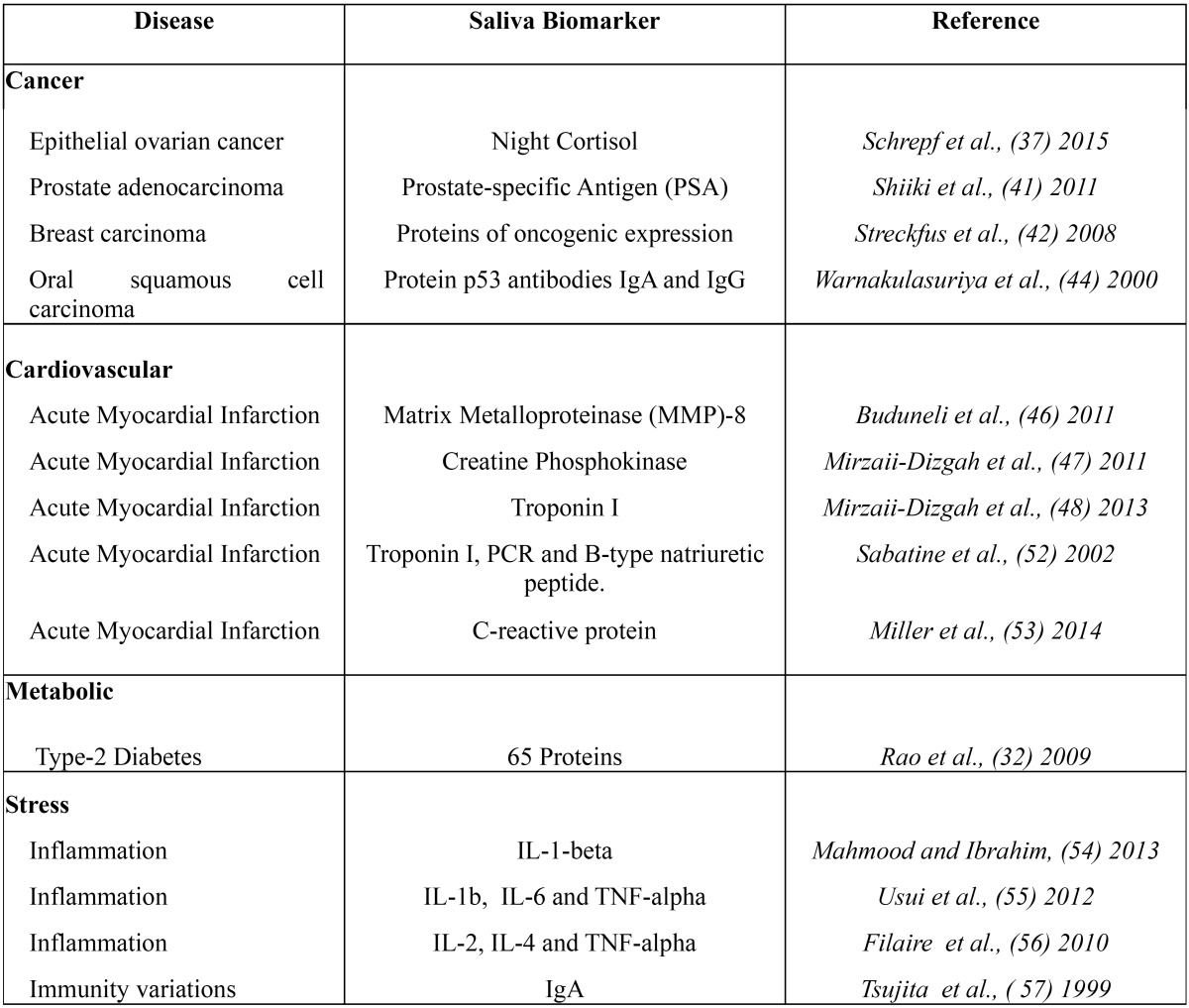
Table of the principal diseases salivary biomarkers.

## Conclusions

• IgA and Cytokines like IL’s and TNF-α & IFN-γ seem to be the most used biomarkers used in diagnostic and prognostic of oral diseases like Periodontitis and caries risk through immunoassays.

• Α-amylase and Total proteins are also two biomarkers commonly utilized in prognostic of oral diseases by ELISA or IFD analysis.

• Provide objective data relating to changes sequentially in the studied concentration of three components of saliva: total protein, immunoglobulin A and alpha-amylase in response to oral surgery, improving the understanding of the pathophysiology of saliva after surgery in the oral cavity.

• Provide criteria for establishing clinical protocols for the treatment of postoperative inflammation in patients undergoing oral surgery, taking into account the changes detected in the concentration of salivary biomarkers studied, thereby contributing to improved quality assistance practiced in the most prevalent oral cavity surgery.
